# Marriage and Birth, Statistics in England

**Published:** 1889-12

**Authors:** 


### MARRIAGE AND BIRTH STATISTICS IN ENGLAND.

It is said in the *Pall Mall Gazette*, that each year 15 people out of every 1,000 marry. Of each 1,000 men who marry 861 are bachelors and 139 are widowers, while of each 1,000 women only 98 have been married before, and 902 are spinsters. Twelve marriages out of every 100 are second marriages. The average age at which men marry is about 27, while the average age at which women marry is about 25 years. Out of every 1,000 persons 602 are unmarried, and 53 widowed. Over one half of all the women between 15 and 55 are unmarried. In all countries about 5 per cent. of marriages prove barren. Among the English nobility 19 per cent. are childless. Married women live two years longer than single ones, although 1 in 70 dies in childbirth. If the mother dies first, the father survives 9½ years; but if the father dies first, the survival of the mother is 11½ years, as an average. Two thousand four hundred and forty-one births occur in England daily, about 33 for each 1,000 inhabitants. February is the month in which the greatest number of births occur, June the month in which occur the fewest. The average number of births for each marriage is 4.33. In every 1,000 births 11 are twins.

